# (Al, Ga)N-Based Quantum Dots Heterostructures on h-BN for UV-C Emission

**DOI:** 10.3390/nano13172404

**Published:** 2023-08-24

**Authors:** Aly Zaiter, Nikita Nikitskiy, Maud Nemoz, Phuong Vuong, Vishnu Ottapilakkal, Suresh Sundaram, Abdallah Ougazzaden, Julien Brault

**Affiliations:** 1 Université Côte d’Azur, Centre National de la Recherche Scientifique (CNRS), Centre de Recherche sur l’Hétéro-Epitaxie et ses Applications (CRHEA), 06560 Valbonne, France; maud.nemoz@crhea.cnrs.fr; 2 CNRS, IRL 2958 Georgia Tech-CNRS, 2 rue Marconi, 57070 Metz, France; pvuong@georgiatech-metz.fr (P.V.); vishnu.ottapilakkal@georgiatech-metz.fr (V.O.); suresh.sundaram@georgiatech-metz.fr (S.S.); abdallah.ougazzaden@georgiatech-metz.fr (A.O.); 3 Georgia Tech-Europe, 2 rue Marconi, 57070 Metz, France; 4 Georgia Institute of Technology, School of Electrical and Computer Engineering, Atlanta, GA 30332-0250, USA

**Keywords:** deep ultra-violet emission, molecular beam epitaxy, nitrides, wide bandgap materials, boron nitride, aluminium gallium nitride, quantum dots, X-ray diffraction, photoluminescence, atomic force microscopy

## Abstract

Aluminium Gallium Nitride (Al_y_Ga_1-y_N) quantum dots (QDs) with thin sub-µm Al_x_Ga_1-x_N layers (with x > y) were grown by molecular beam epitaxy on 3 nm and 6 nm thick hexagonal boron nitride (h-BN) initially deposited on c-sapphire substrates. An AlN layer was grown on h-BN and the surface roughness was investigated by atomic force microscopy for different deposited thicknesses. It was shown that for thicker AlN layers (i.e., 200 nm), the surface roughness can be reduced and hence a better surface morphology is obtained. Next, Al_y_Ga_1-y_N QDs embedded in Al_0.7_Ga_0.3_N cladding layers were grown on the AlN and investigated by atomic force microscopy. Furthermore, X-ray diffraction measurements were conducted to assess the crystalline quality of the AlGaN/AlN layers and examine the impact of h-BN on the subsequent layers. Next, the QDs emission properties were studied by photoluminescence and an emission in the deep ultra-violet, i.e., in the 275–280 nm range was obtained at room temperature. Finally, temperature-dependent photoluminescence was performed. A limited decrease in the emission intensity of the QDs with increasing temperatures was observed as a result of the three-dimensional confinement of carriers in the QDs.

## 1. Introduction

The Minamata Convention, convened in Japan on 10 October 2013, led to the establishment of an international treaty aimed at restricting the use of mercury (Hg) and mercury-based devices. This treaty paved the way for the advancement of more efficient and environmentally friendly light sources, such as light-emitting diodes (LEDs) [1]. LEDs provide numerous benefits including compact size, a wide range of wavelength emissions, extended lifetimes, and reduced power consumption over commonly used Hg lamps that are defined by being bulky, suffer from high voltages, and pose toxicity risks and recycling issues [2]. LEDs based on aluminum gallium nitride ((Al, Ga)N) can emit light in the ultraviolet (UV) range, particularly in the deep UV (DUV) or UV-C range, with emission wavelengths below 280 nm. These DUV emissions are achievable with Al compositions generally exceeding 40%. This particular aspect has attracted wide interest in developing (Al, Ga)N-based LEDs since the UV-C region is considered the germicidal range of UV radiation: in this range, the damaging effect on intercellular components (e.g., DNA, RNA, and proteins) of microbes occurs, which can be applied in strategic applications such as surface disinfection, sterilization, and water purification [3,4]. Despite the significant amount of research conducted in the past ten years, (Al, Ga)N UV LEDs with a wavelength shorter than 365 nm exhibit a low external quantum efficiency (EQE) that typically falls within the single-digit percentage range. Despite recent advances that have increased the efficiency to a maximum value of 20% for UV-C LEDs emitting around 275 nm [5], the EQE typically remains below 10%. The EQE of LEDs is determined by the product of injection efficiency (IE), light extraction efficiency (LEE), and internal quantum efficiency (IQE). Hence, new approaches and methods are required to enhance their efficiency values.

Sapphire is predominantly utilized as the preferred substrate for the growth of (Al, Ga)N-based LEDs since it offers several advantages such as its low cost, large size availability (up to 8-inch wafers), and its transparency in the UV range. Nevertheless, growing on sapphire presents several drawbacks. The high lattice mismatch (~13% for AlN and a_AlN_ = 3.112 Å for the basal plane lattice parameter) and the mismatch in the lattice thermal coefficient (~43% for the basal plane) negatively impact the crystalline structural quality of the epitaxial layers, resulting in increased threading dislocation densities (TDDs). These TDDs act as non-radiative recombination centers, causing a decrease in the IQE and the EQE. Therefore, favoring radiative recombination by improvement of the carrier confinement into the active region can increase the IQE in LEDs. The growth of three-dimensional (3D) quantum dots (QDs) as the active region instead of 2D quantum wells (QWs) can help reduce the impact of non-radiative recombination of excitons with surrounding TDs, thus increasing the IQE. This is attributed to the better carrier confinement offered by the three-dimensional confinement potential of QDs, resulting in a higher probability of radiative recombination. The use of two-dimensional (2D) materials has also gained widespread attention as a potential solution to overcome the issue of lattice mismatch and the high density of the threading dislocations [6]. The extended crystalline planar structures of 2D materials are characterized by robust in-plane covalent bonds and relatively weaker out-of-plane van der Waals forces. This unique bonding arrangement facilitates the straightforward extraction of individual layers by breaking the van der Waals bonds, causing minimal damage to both the extracted layer and the remaining structure [7]. This unique feature of 2D materials enables van der Waals epitaxy to exploit them, greatly reducing the lattice mismatch of conventional heterostructure growth methods [6]. Among the various options of 2D materials, hexagonal boron nitride (h-BN) stands out as highly suitable due to its chemical compatibility with AlN or (Al, Ga)N-based epitaxial layers. Moreover, h-BN templates can serve as mechanical release layers, enabling the transfer of nitride-based LEDs to suitable substrates and thereby facilitating the development of flexible devices [8]. However, growing high-quality III-nitride films on h-BN is challenging due to the lack of dangling bonds on the surface, which complicates the nucleation step, as seen in the formation of randomly oriented, polycrystalline, isolated islands in gallium nitride (GaN) growth on h-BN [8]. AlN, on the other hand, with its lower mobility and higher sticking coefficient of Al adatoms, may serve as a more favorable nucleation layer on h-BN [9,10].

Research on MBE growth of (Al, Ga)N heterostructures on h-BN is limited. Our group recently used face-to-face high-temperature annealing (FFA) to enhance the crystalline quality and surface morphology of AlN layers grown by MBE on h-BN/sapphire templates [11]. Previous studies on nitride heterostructures grown on h-BN mainly used metal–organic vapor phase epitaxy (MOVPE) growth modes. In their study, Qingqing Wu et al. conducted the growth of AlN and deep ultraviolet (DUV) LEDs on monolayer h-BN, which was transferred onto a sapphire substrate after being grown via low-pressure chemical vapor deposition (CVD) on a copper (Cu) foil substrate [12]. The same research group also reported the successful development of crack-free crystalline AlN and DUV LEDs emitting at 281 nm on multilayer h-BN grown by MOVPE [13]. These findings highlight the advantages of utilizing multilayer h-BN for achieving high-quality epilayers and devices on large surfaces. J. Shin et al. reported the fabrication of vertical full-colormicro-LEDs using 2D material-based layer transfer techniques, allowing for the mechanical transfer of LED layers from the 2D materials and the reuse of the substrate [14]. However, MBE growth of QD-based (Al, Ga)N heterostructures on h-BN for UV-C flexible LED fabrication has not been reported yet and was the subject of investigation in this study.

This study focused on the growth of AlN, Al_x_Ga_1-x_N thick layers, and Al_y_Ga_1-y_N QD layers using MBE on h-BN/sapphire templates. Additionally, the impact of increasing the thickness of h-BN layers on the surface morphology of the AlN epitaxial layers was investigated. The h-BN layers with thicknesses of 3 nm and 6 nm were directly grown on 2-inch sapphire substrates using MOVPE. The impact of h-BN on the AlN/Al_x_Ga_1-x_N layer’s growth was investigated, showing two different orientations in the growth plane. Finally, the Al_y_Ga_1-y_N QDs structural and optical properties were studied, showing a homogenous distribution of QDs and a successful emission below 280 nm in the UV-C, thus paving the way for the fabrication of flexible QD based Al_x_Ga_1-x_N UV-C LEDs.

## 2. Materials and Methods

**Figure 1:**
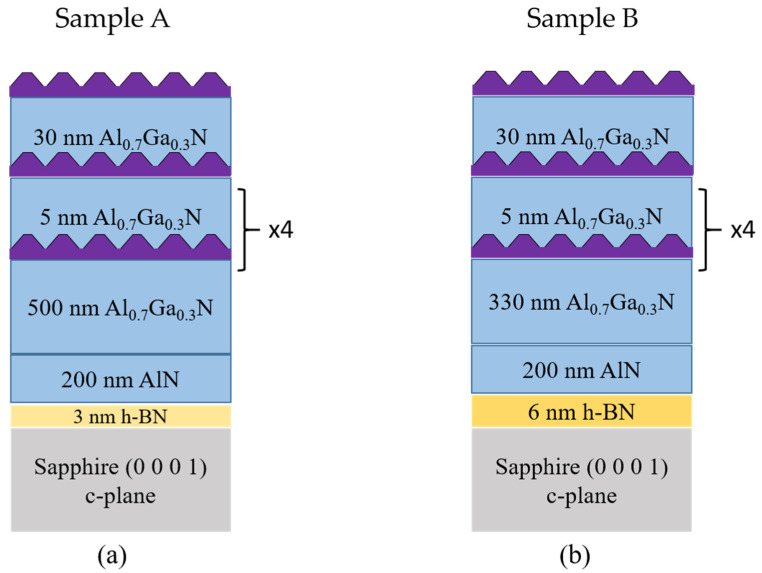
Schematics of the two Al_y_Ga_1-y_N quantum dot (QDs) structures grown on h-BN/sapphire templates. (**a**) Al_y_Ga_1-y_N QDs structure grown on 3 nm h-BN on sapphire (sample A). (**b**) Al_y_Ga_1-y_N QDs structure grown on 6 nm h-BN on sapphire (sample B).

The growth of h-BN templates with thicknesses of 3 nm and 6 nm was performed using an Aixtron MOVPE close-coupled showerhead (CCS) reactor on (0 0 0 1) sapphire substrates. The process was carried out at a temperature of 1280 °C and a pressure of 90 mbar. Triethylboron (TEB) and ammonia (NH_3_) were employed as precursor gases for boron (B) and nitrogen (N), respectively. More comprehensive information on the growth conditions for h-BN can be found in previously published reports [15]. The growth of Al_x_Ga_1-x_N/AlN structures on the h-BN templates was performed using MBE in a RIBER 32P reactor. Solid sources of the III-elements (Al, Ga) and NH_3_ as a nitrogen source were utilized, except for the QD active region where NH_3_ was replaced by a nitrogen (N_2_) plasma source. This substitution was necessary to enable the formation of QDs and 3D islands, as NH_3_ would result in a 2D growth mode that inhibits their formation [16]. Under N_2_, a 2D–3D “Stranski–Krastonov” growth mode can be achieved [16,17], allowing for the growth of Al_y_Ga_1-y_N QDs on Al_x_Ga_1-x_N (with x > y). Two structures, known as sample A and sample B, were fabricated (see Figure 1). The fabrication process for both samples was similar, except for the thickness of the Al_x_Ga_1-x_N layer. For both samples, a 200 nm thick AlN layer was initially grown with the following conditions: a 10 nm thick AlN buffer layer was grown at 1070 °C with an ammonia flow rate of 50 sccm and a growth rate of 50 nm/h. Subsequently, the 200 nm AlN layer was grown at 1120 °C with an ammonia flow rate of 50 sccm and a growth rate of 100 nm/h. Following this, a 500 nm thick Al_0.7_Ga_0.3_N layer was grown at 870 °C for sample A and a 330 nm thick layer for sample B, both at a growth rate of 290 nm/h. Next, an active region consisting of six Al_0.3_Ga_0.7_N QD planes with an Al nominal composition (n.c.) of 0.3, separated by Al_0.7_Ga_0.3_N barriers lattice matched to the Al_0.7_Ga_0.3_N template, was deposited. The equivalent 2D thickness of the Al_0.3_Ga_0.7_N (n.c.) QDs was 7 monolayers (MLs), approximately 1.8 nm, with 1 ML corresponding to half the c-lattice parameter, considering a variation of the lattice parameter following Vegard’s law between AlN and GaN. In between the QD planes, 5 nm thick Al_0.7_Ga_0.3_N cladding layers were grown for both samples. After the deposition of the fifth QD plane, a 30 nm thick Al_0.7_Ga_0.3_N layer was grown at 820 °C. Finally, the sixth and last QD plane was deposited on the surface of the top cladding layer for both samples.

Atomic force microscopy (AFM) EDGE-DIMENSION (BRUKER, Billerica, MA, USA), operating in tapping mode with a silicon tip with a radius between 5 and 10 nm, was used to study the surface morphologies of h-BN and AlN. In addition, a diamond-coated tip with a typical radius between 5 and 10 nm was also used to investigate the morphology of the surface QDs plane, and all data were processed using WSxM software [18]. Furthermore, X-ray diffraction (XRD) measurements were conducted using a PANalytical X’Pert PRO MRD four-circle diffractometer (Malvern Panalytical, Malvern, United Kingdom) to assess the crystalline quality of the Al_x_Ga_1-x_N/AlN layers and examine the impact of h-BN on the subsequent layers. Regarding the optical characteristics, continuous wave PL measurements were carried out at room temperature (RT) and low temperature (LT), i.e., at 300 K and 12 K, in a closed-cycle Helium (He) cryostat using a frequency-doubled Argon (Ar) laser at 244 nm (5.08 eV) with an excitation power of 20 mW.

## 3. Results

In the first part of this section, the growth of AlN on h-BN templates and the study of its surface roughness evolution as a function of the AlN thickness are presented. The second part is devoted to an in-depth XRD characterization of Al_0.7_Ga_0.3_N, as well as Al_0.3_Ga_0.7_N (n.c.) QDs’ main structural and optical properties, including a surface morphology study by AFM and PL measurements.

### 3.1. AlN Growth by MBE

#### 3.1.1. Characterization of h-BN Templates before Growth

**Figure 2:**
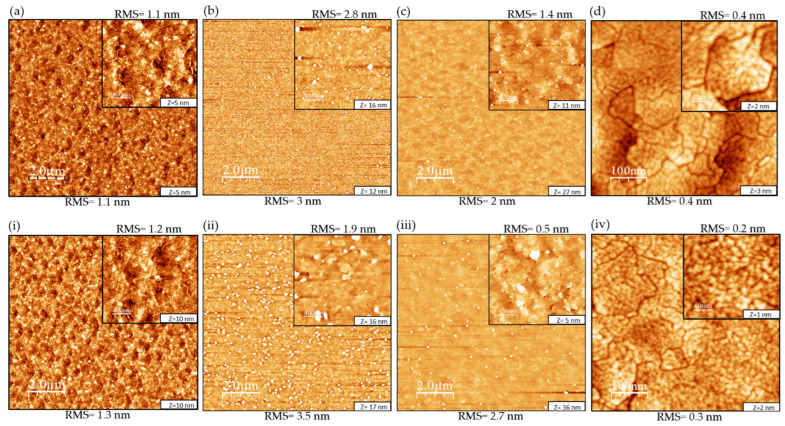
Atomic force microscopy images of (**a**) 3 nm thick h-BN layer grown on sapphire c-plane substrates, (**b**) 50 nm AlN grown on 3 nm h-BN, (**c**) 200 nm AlN grown on 3 nm h-BN, (**d**) Al_0.3_Ga_0.7_N QDs morphology on the surface of sample A, (**i**) 6 nm thick h-BN layer grown on sapphire c-plane substrates, (**ii**) 50 nm AlN grown on 6 nm h-BN, (**iii**) 200 nm AlN grown on 6 nm h-BN, and (**iv**) Al_0.3_Ga_0.7_N QD morphology on the surface of sample B. (**a**–**c**,**i**–**iii**) are (10 × 10) μm^2^ images and the inset shows (2 × 2) μm^2^ scan images, while (**d**,**iv**) are (500 × 500) nm^2^ scan images with an inset of (200 × 200) nm^2^ scan images. The term Z represents the vertical scale and the variation in height in the AFM images, and its value is reported at the bottom right-hand side of each image.

To analyze the surface morphology, the 3 nm and 6 nm thick h-BN layers on sapphire samples were initially examined. AFM topographic images of (10 × 10) µm^2^ and (2 × 2) µm^2^, along with their corresponding root-mean-square (RMS) values, are depicted in Figure 2a,i.

Figure 2a is a (10 × 10) µm^2^ AFM scan that shows the 3 nm h-BN layer covering the entire wafer surface with a measured surface RMS roughness of 1.1 nm. The inset is a (2 × 2) µm^2^ AFM scan with a surface RMS roughness measured of 1.1 nm. Figure 2i instead shows the 6 nm h-BN layer with a surface RMS roughness of 1.3 nm. The inset of the (2 × 2) µm^2^ scan is similar to the one in Figure 2a with a surface RMS roughness of 1.2 nm. The occurrence of wrinkles, represented by the white segments observed in the AFM images, is a common characteristic of 2D materials. Regarding h-BN, these wrinkles are primarily caused by the variation in the thermal expansion coefficient (TEC) between h-BN and sapphire substrates. During the cooling process, this TEC mismatch leads to the generation of compressive strain within the h-BN layer, resulting in the formation of wrinkles. The wrinkling instability facilitates the release of energy, resulting in the creation of surface roughness in the sample [19]. 

#### 3.1.2. AlN Growth on h-BN/Sapphire Templates by MBE

We proceeded to grow AlN (total thickness of 200 nm) on both the 3 nm and 6 nm thick h-BN/sapphire templates by MBE. The 200 nm layers were grown in three steps. First, a 50 nm layer was grown followed by another 50 nm thick layer and a 100 nm final layer. This systematic growth was carried out as a way to study the evolution of the AlN layer growth on h-BN in terms of surface morphology with increasing thickness. Figure 2b,c,ii,iii illustrate the surface morphology evolution when the AlN thickness increased from 50 nm to 200 nm for both samples. For sample A, the initial surface morphology of the 50 nm thick layer was a rough one with an RMS = 3 nm at a (10 × 10) µm^2^ scan range. It was dominated by a high density (~1.7 × 10^9^ cm^−2^) of 3D island-like structures (height ~32 nm ± 14 nm and lateral size ~119 nm ± 30 nm). After the thickness was increased to 200 nm, the surface morphology became rougher with an RMS increase to 3.5 nm. On the other hand, the density of the 3D island-like structures decreased to ~5.4 × 10^8^ cm^−2^ as well as its height (~23 nm ± 8 nm) but its lateral size increased (~152 nm ± 40 nm). In addition, the surface morphology in-between the islands, observed in the (2 × 2) µm^2^ scan range inset image in Figure 2ii, showed a smoother surface compared to the (10 × 10) µm^2^ scan range with an RMS = 1.9 nm. Regarding sample B, the initial surface morphology of the 50 nm thick layer was also rough with an RMS = 2 nm on a (10 × 10) µm^2^ scan range. It was also dominated by 3D island-like structures (density ~3 × 10^8^ cm^−2^, height ~22 nm ± 7 nm, and size ~84 nm ± 16 nm). After the thickness was increased to 200 nm, the surface roughness also increased with an RMS = 2.7 nm, similar to sample A’s surface evolution. The island’s density decreased to ~9.1 × 10^7^ cm^−2^ but its height and lateral size increased (~40 nm ± 15 nm and 124 nm ± 20 nm). The surface morphology in-between the islands observed in the (2 × 2) µm^2^ scan range inset image in Figure 2iii showed a very smooth surface compared to the (10 × 10) µm^2^ scan range with an RMS = 0.5 nm. With the increase in AlN thickness, the RMS roughness of the surface improved positively for both samples. Initially, the increase in AlN thickness resulted in a reduction in island density, although the individual islands grew larger in size. As a result, the surface between the islands became smoother, contributing to the improved RMS roughness. We can still see a difference between the AlN growth on the 3 nm and 6 nm thick h-BN templates where smoother AlN layers with lower islands density were obtained on the 6 nm h-BN compared to the 3 nm h-BN template despite both h-BN templates having the same initial surface RMS roughness values of 1.2 nm.

### 3.2. Al_0.3_Ga_0.7_N/Al_0.7_Ga_0.3_N QDs Structural and Optical Properties

#### 3.2.1. Morphological Properties 

After the growth of the Al_0.7_Ga_0.3_N layers and Al_0.3_Ga_0.7_N QD active region, AFM was performed in order to investigate the QDs’ structural properties. Figure 2d,iv show AFM images of samples A and B for (500 × 500) nm^2^ and (200 × 200) nm^2^ scan ranges. 

**Figure 3:**
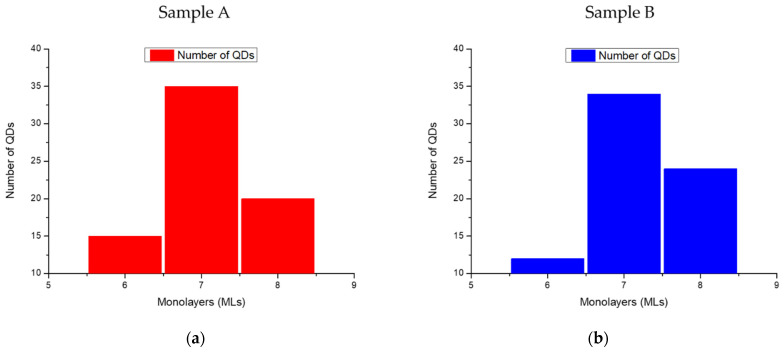
Histograms showing the QDs’ height distributions as a function of monolayer (ML) units (1 ML = 0.257 nm) for (**a**) sample A and (**b**) sample B.

The QDs’ densities, heights, and diameters were determined from AFM measurements for both samples. For sample A, the QDs’ average height was found to be ranging between 6 MLs and 8 MLs with the highest QDs population having a height of 7 MLs based on the histogram (red sticks) in Figure 3a. The QDs’ average diameter was found around 13 nm ± 3 nm. Furthermore, for sample B, the QDs’ height was also found to be ranging between 6 MLs and 8 MLs based on the histogram (blue sticks) in Figure 3b, with the highest number for 7 MLs and the QDs average diameter around 15 nm ± 2 nm. The QDs’ height results for both samples are similar to previous results obtained by our group on Al_y_Ga_1-y_N QDs grown by MBE [20]. Regarding the QDs’ density, it was estimated at 4 × 10^11^ cm^−2^ for both samples. This trend of high QDs density (>10^11^ cm^−2^) is usually observed in the case of Al_y_Ga_1-y_N QDs compared to GaN QDs [20], which is attributed to the lower surface mobility characteristic of Al adatoms compared to Ga ones. Meanwhile, both samples presented domains on their surface, which are separated by surface depressions (depression depth for both samples ~8 Å) with a density ranging from around 3 × 10^7^ to 4 × 10^7^ cm^−1^. XRD measurements have indicated that the (Al, Ga)N structures are experiencing a tensile strain at room temperature (not shown). A thermal expansion difference between h-BN and AlN could be at the origin of a tensile strain, contrary to the compressive strain typically observed during the growth of AlN/AlGaN on sapphire. Therefore, strain effects could be at the origin of the specific surface morphology of Al_0.7_Ga_0.3_N layers, related to the interplay between sapphire, h-BN, and the (Al, Ga)N materials, and the formation of the surface depressions, the study of which goes beyond the scope of this work. 

#### 3.2.2. Crystal Properties by X-ray Diffraction 

**Figure 4:**
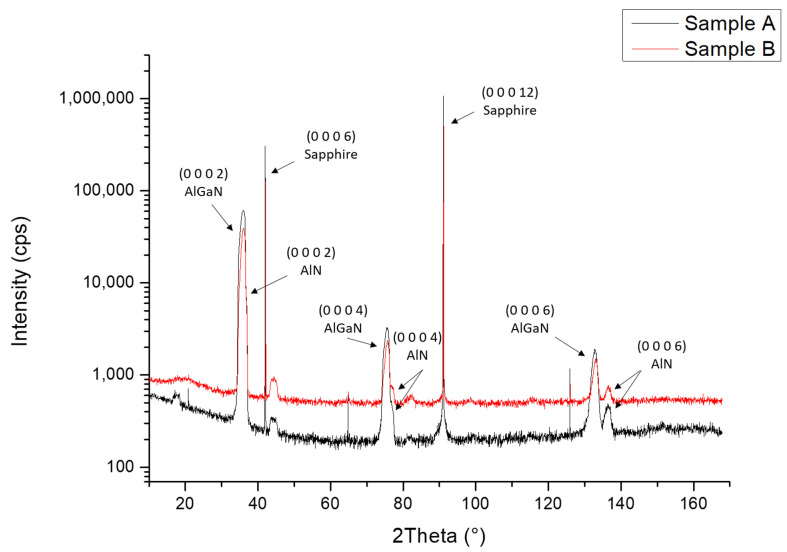
XRD 2θ-ω scan for samples A and B.

Figure 4 illustrates the 2θ-ω X-ray diffraction diagram performed on samples A and B ranging from 10° to 170° in order to study all the layers‘’ orientations along the growth direction.

For both samples, only the (0 0 0 1) orientation was observed for nitride layers: at 36° the Al_x_Ga_1-x_N and AlN (0 0 0 2) peaks were mingled. The Al_x_Ga_1-x_N and AlN (0 0 0 4) peaks were seen at 75.3° and 76.5°, respectively. The Al_x_Ga_1-x_N and AlN (0 0 0 6) peaks were well separated at 132.9° and 136.38°. The thinner peaks correspond to the sapphire substrate: the (0 0 0 6) and (0 0 0 12) reflections were observed with high intensities and three forbidden reflections of sapphire were detected. It should be mentioned that two peaks, observed at 17.8° and 44.4°, came from the sample holder itself since the studied samples are smaller than the X-ray beam footprint. 

**Figure 5:**
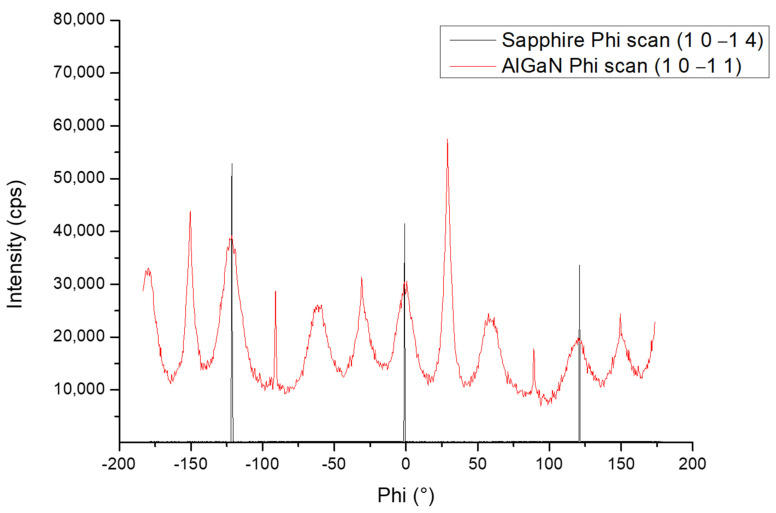
XRD Phi scan performed on sample A along the (1 0 −1 1) and the (1 0 −1 4) skew planes for Al_0.7_Ga_0.3_N and sapphire, respectively.

Figure 5 illustrates the XRD Phi (in-plane rotation of the sample) scans of the (1 0 −1 1) Al_0.7_Ga_0.3_N layer and (1 0 −1 4) sapphire substrate performed on sample A. Twelve peaks were observed for the Al_0.7_Ga_0.3_N layer instead of the six peaks expected for a hexagonal structure. This can be explained by the presence of two domains twisted by 30° in the growth plane.

When (Al, Ga)N hexagonal layers are grown directly on sapphire, the (Al, Ga)N unit cell is turned by an angle of 30° compared to that of sapphire. Thereby, the sharp peaks have the classic orientation of (Al, Ga)N on sapphire. The wide peaks represent a new in-plane orientation turned by 30°, thus creating two different orientations in the growth plane. The h-BN layer permitted this structural arrangement since it is forbidden for such orientations to happen on sapphire.

**Figure 6:**
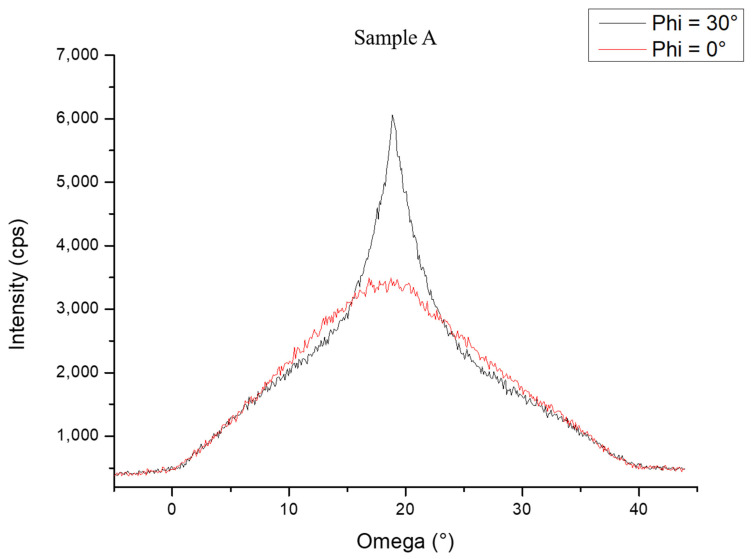
XRD omega scan along the AlGaN (1 0 −1 1) skew plane for sample A chosen from two different Phi peaks (0° and 30°).

The study continued on the Al_0.7_Ga_0.3_N (1 0 −1 1) plane by performing two omega scans at Phi = 0° and Phi = 30°, respectively. The two omega scans showed different intensities and FWHM values, as illustrated in Figure 6. The ω scan peak in red color (Phi = 0°), corresponds to the Al_0.7_Ga_0.3_N orientation due to the presence of h-BN on the sapphire surface. The FWHM of this peak is equal to 20°, indicating crystalline domains with a high defect density. On the other hand, the ω scan peak in black color (Phi = 30°) corresponds to the Al_0.7_Ga_0.3_N orientation typically observed on sapphire. However, it presented an unusual peak shape: a careful analysis shows that this peak was composed of a base similar to the large peak presented in red color (Phi = 0°). This feature suggests that in this classical orientation of Al_0.7_Ga_0.3_N, two domains are present. One of which, corresponding to the thinner part of the peak (FWHM = 7°), was of better crystalline quality than the domain corresponding to the wider part of the peak (FWHM = 20°).

#### 3.2.3. Optical Properties

- Continuous wave photoluminescence

**Figure 7:**
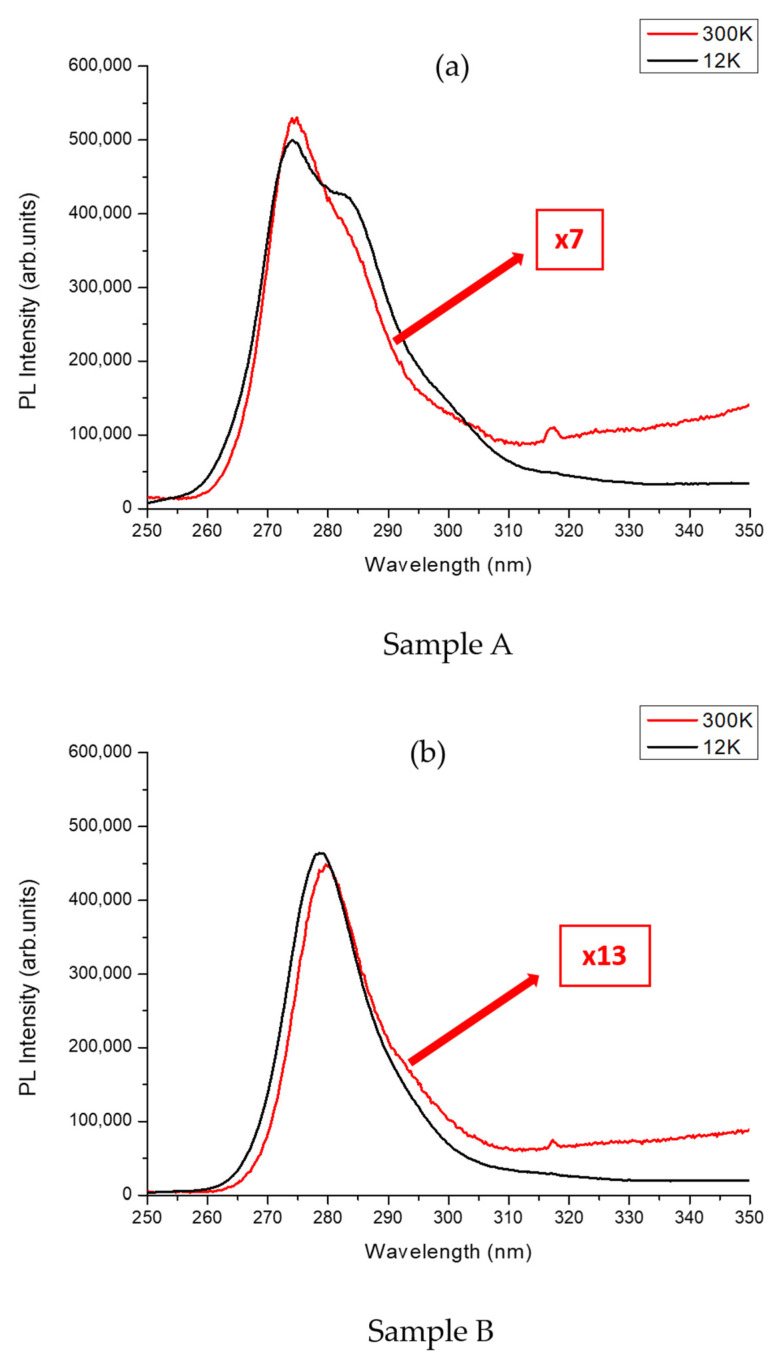
Photoluminescence (PL) spectrum of Al_0.3_Ga_0.7_N QDs with an Al_0.3_Ga_0.7_N deposited amount of 7 MLs at 12 K and 300 K for samples A and B. (**a**) The PL intensity has been multiplied by 7 for the spectrum obtained at 300 K. (**b**) The PL intensity has been multiplied by 13 for the spectrum obtained at 300 K.

In order to study the optical characteristics of the Al_0.3_Ga_0.7_N QDs, continuous PL measurements at LT (12 K) and at RT (300 K) were performed and compared for both samples using the same excitation conditions. Figure 7 shows the PL spectrum at 12 K and 300 K for both samples.

For sample A with a QD nominal composition y of 0.3, two PL peak emissions were observed at LT. The main PL peak with the highest intensity had an emission in the UV-C region at 275 nm (4.5 eV). It originates from the exciton radiative recombination in the Al_0.3_Ga_0.7_N QDs. In addition to this main PL peak, a low energy shoulder peak at 283 nm (between 4.3 eV and 4.4 eV) with a lower intensity was also observed. The modulation of PL intensity is affected by Fabry–Perot interference fringes caused by the significant differences in refractive indices at the interfaces of (Al, Ga)N/sapphire and air/(Al, Ga)N. As shown in Figure 7a, a weak decrease in both the QDs’ PL peaks was observed from LT to RT, confirming the strong 3D carrier confinement in the Al_0.3_Ga_0.7_N QDs. The FWHM of the PL peak at RT was 18 nm. 

On the other hand, sample B, with the same QD nominal composition y of 0.3 as sample A, had one clear PL peak emission observed at both LT and RT in the UV-C region at a very close wavelength of 280 nm (4.43 eV). As shown in Figure 7b, a decrease in the QDs’ PL peak intensity was observed from LT to RT. The FWHM of the PL peak at RT was 16 nm, a value roughly similar to sample A. 

From these measurements, it was found that the spectrally integrated PL intensity ratio (I (300 K)/I (12 K)) of the QD emission peak between RT and LT was roughly 10%. This is rather high compared to the case of QWs on high-dislocation-density (Al, Ga)N materials, which is below 10^−2^ [21]. In addition, in comparison with a reference Al_y_Ga_1-y_N QDs/Al_x_Ga_1-x_N structure directly grown on AlN/sapphire, the ratio of the PL integrated intensities was found to be between 15 and 45 times lower for Al_y_Ga_1-y_N QDs grown on h-BN (see Supplementary Material). This result indicates that there is still room for improvement in the structural design and growth condition optimizations of both h-BN and (Al, Ga)N materials for UVC LEDs.

## 4. Discussion

The proposed study, which focused on both 3 nm and 6 nm h-BN thicknesses, aimed to explore the impact of such h-BN templates on the nucleation and growth of (Al, Ga)N-based heterostructures and quantum dot (QD) active regions, targeting UVC emitters. Therefore, the choice of these specific thicknesses ensured complete coverage of h-BN on the sapphire substrate, enabling both efficient nucleation of the AlN layer and opening the perspective of a possible subsequent exfoliation of the layer, as was already reported in previous study by Prof. Ougazzaden’s group [22]. By employing ammonia-based MBE, the growth of AlN on h-BN/sapphire templates resulted in the formation of Al-polar layers characterized by predominantly 2D surfaces. However, the presence of 3D islands disrupted the otherwise flat morphology of the layers. Following the subsequent AlN layer growth and thickness increase from 50 nm to 200 nm in total, the surface RMS roughness estimated by AFM measurements for a large (10 × 10) µm^2^ scan range increased for both samples, while it decreased for a small (2 × 2) µm^2^ scan range. This feature originates from the presence of large 3D AlN islands originating from an initial 3D growth mode (see Supplementary Material), which have also been observed for AlN growth on sapphire [23], and lead to an important increase in the surface RMS roughness. As the AlN layer thickness was increased and the 3D island’s density was decreased, the surface RMS roughness at a smaller scale was reduced, consequently leading to low RMS values for (2 × 2) μm^2^ scans in-between the 3D islands. After the growth of the Al_0.7_Ga_0.3_N layers and Al_0.3_Ga_0.7_N QDs active region, AFM measurements showed a homogenous distribution of QDs with the majority of QDs having a height of 7 MLs and a density around 4 × 10^11^ cm^−2^ for both samples. In addition, the XRD symmetric 2θ-ω scan of samples A and B showed that all the layers observed in both samples were grown along the growth axis (c-axis). On the other hand, the XRD 360° Phi scan for sample A showed twelve peaks instead of the usual six peaks observed on Al_x_Ga_1-x_N structures grown on sapphire. After performing a Phi scan on the sapphire template, it was found that the six other peaks actually came from another domain that was rotated in the growth plane by an angle of 30°, compared to the domain that originated from the wurtzite crystal structure of the (Al, Ga)N layers grown on sapphire. This is due to the h-BN template that created two different orientations in the growth plane. This result confirms the orientation guidance from h-BN observed in a previous study carried out by S. Sundaram et al. where III-nitrides were grown on h-BN on c-plane sapphire [24]. They observed that the varying degrees of misorientation that can be observed in III-nitrides are depending on the crystalline quality of h-BN. The III-nitrides could be even amorphous beyond a certain crystallinity limit of h-BN. This observation shows the importance of growing good crystalline quality h-BN layers.

QDs’ PL emissions showed a maximum intensity in the UV-C range between 275 and 280 nm at room temperature and with an FWHM around 16–18 nm, which is attributed to fluctuations in both the QDs’ size and composition (Al concentration) [25].

In our study, the structural properties of (Al, Ga)N layers grown on h-BN were compared to those obtained in a previous study conducted by our research group wherein we investigated the growth of Al_x_Ga_1-x_N/Al_y_Ga_1-y_N QDs on sapphire using MBE. Based on X-ray measurements, it was found that the FWHM of the Al_x_Ga_1-x_N layers grown on sapphire [20] was significantly narrower compared to the FWHM of the Al_x_Ga_1-x_N layers grown on h-BN. However, and importantly, the temperature dependence of the PL integrated intensity of Al_0.3_Ga_0.7_N QDs/Al_0.7_Ga_0.3_N on sapphire was observed to be similar to that of Al_0.3_Ga_0.7_N QDs/Al_0.7_Ga_0.3_N on h-BN, showing a PL integrated intensity ratio between 10K and 300K of the same order of magnitude [20]. Put together, these results suggest that while there may be significant differences in the structural properties of heterostructures grown on sapphire or on h-BN templates, the temperature-dependent behavior and overall PL characteristics of UVC-emitting Al_0.3_Ga_0.7_N QDs/Al_0.7_Ga_0.3_N active regions remain comparable. We propose that the temperature dependence of the PL integrated intensity of Al_y_Ga_1-y_N QDs is not strongly influenced by the crystalline quality of the material, and this can be attributed to the robustness of the QDs resulting from their 3D confinement of excitons at the nanoscale. It is worth noting that variations in PL characteristics can still be observed among different samples, including those grown directly on sapphire, but with a maximum magnitude limited to 10 only when comparing the most and less radiatively efficient samples (see Supplementary Material). 

## 5. Conclusions

Al_0.3_Ga_0.7_N QD-based structures emitting in the UV-C range were grown on h-BN/sapphire templates by MBE using two different h-BN thicknesses: 3 nm and 6 nm. The structural properties of Al_0.7_Ga_0.3_N layers were studied, showing an impact of the h-BN layers on the subsequent structures in the form of an orientation guidance and creating two different orientations in the growth plane affirming the crucial importance of growing high-crystalline-quality h-BN layers. Optical characterizations showed that the QDs for sample A and sample B had emissions in the UV-C with a maximum intensity emission ranging between 275 nm (4.5 eV) and 280 nm (4.43 eV). In addition, the spectrally integrated PL intensity ratio (I (300K)/I (12K)) of the QD emission peak between RT and LT for samples A and B was determined to be around 10%. This study demonstrated the successful growth of UV-C-emitting Al_0.3_Ga_0.7_N QDs structures by MBE on h-BN/sapphire templates. This result will enable us to move to the next step, which is the fabrication of QD-based UV-C LEDs and their exfoliation, hence demonstrating the possible fabrication of flexible UV-C LEDs.

## Data Availability

All data included in this study are available upon request by contact with the corresponding author.

## Supplementary Materials

The following supporting information can be downloaded at: https://www.mdpi.com/article/10.3390/nano13172404/s1, Figure S1: Reflection high-energy electron diffraction (RHEED) images, along the <1 − 100> direction, of the AlN growth on h-BN/sapphire templates; Figure S2: RMS roughness values evaluated as a function of the AlN thickness for samples A and B; Figure S3: Photoluminescence measurements of Al_y_Ga_1-y_N QD plane at room temperature (300 K) for different samples; Table S1: Wavelength emission and PL integrated intensity ratio between LT and RT for different Al_y_Ga_1-y_N QDs/Al_x_Ga_1-x_N structures grown on sapphire and on h-BN; Table S2: X-ray rocking curve diffraction measurements of (0 0 0 2) and (1 0 −1 3) symmetric and asymmetric reflections for Al_0.7_Ga_0.3_N structures grown on sapphire and Al_0.7_Ga_0.3_N structures grown on h-BN/sapphire.
