# Supplementary material for: (Al, Ga)N-Based Quantum Dots Heterostructures on h-BN for UV-C Emission
Source: Nanomaterials (Basel). 2023 Aug 24;13(17):2404. doi: 10.3390/nano13172404 (PMC10489961; doi:10.3390/nano13172404)
Supplement: Supplementary file 1 [file nanomaterials-13-02404-s001.zip › nanomaterials-2492342-supplementary.pdf]

Supplementary material:

1. Growth of AlN on h-BN / Sapphire by MBE

By using ammonia-based molecular beam epitaxy (MBE), the growth of AlN on h-BN/sapphire templates resulted in the growth of Al-polar layers with predominantly two-dimensional (2D) surfaces, which were accompanied by the presence of three-dimensional (3D) islands, as illustrated in figure S1(a). As the subsequent AlN layers were grown and their thicknesses increased from 50 nm to 200 nm, a transition in the reflection high-energy electron diffraction (RHEED) pattern was observed, shifting from an initial 3D growth mode to a 2D growth mode, as depicted in figure S1(b). This transition was characterized by a decrease in the density of 3D islands and a reduction in the root mean square (RMS) roughness at smaller length scales. Consequently, when performing (2 × 2) μm^2 scans in the areas between the 3D islands, low RMS values below 1 nm were obtained.

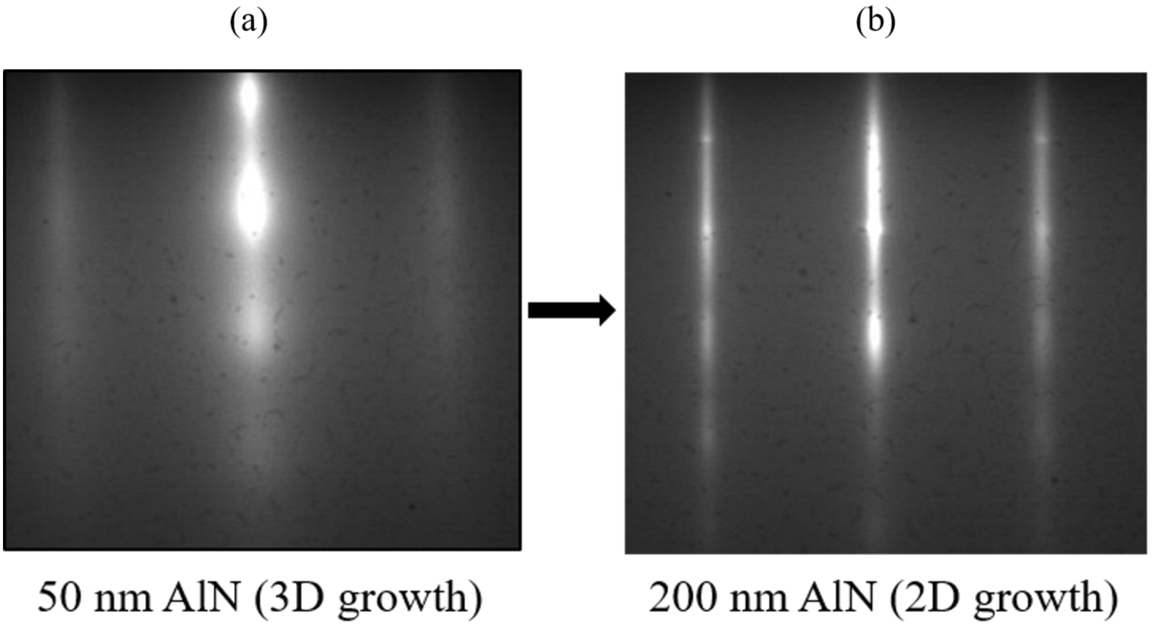

Figure S1. Reflection high-energy electron diffraction (RHEED) images, along the <1-100> direction, of the AlN growth on h-BN / sapphire templates. (a) For 50 nm thick AlN layer, which surface morphology is defined by Bragg spots indicating the presence of 3D islands on the surface. (b) For a 200 nm thick AlN layer that is mainly characterized by diffraction lines, indicating a smoother 2D surface morphology. This modification of the RHEED pattern comes from the smoothening of the surface morphology and the reduction 3D islands density as the AlN layer thickness increases.

2. RMS roughness evolution as a function of the AlN thickness for samples A and B

Figure S2 illustrates the RMS roughness evolution as a function of the AlN thickness for samples A and B. The RMS values are measured on (10 x 10) µm² and (2 × 2) µm² atomic force microscopy topographic images.

For the 3 nm h-BN template, along the (10 x 10) µm² scan range, the initial growth at 50 nm had a surface RMS roughness of 3 nm that increased to 3.5 nm after a total growth of 200 nm. On the other hand, the RMS variation along the (2 x 2) µm² scan range was de-creased from 2.8 nm for the initial growth of 50 nm to 1.9 nm after the total AlN growth of 200 nm. Regarding the 6 nm h-BN template, the RMS was 2 nm for the initial growth, which is lower compared to the 50 nm AlN on 3 nm h-BN template. Following the total growth of a 200 nm thick layer, the RMS increased to 2.7 nm. On the contrary, the RMS variation along the (2 x 2) µm² scan range was also decreased, similarly to sample A (2 x 2) µm² scan range RMS evolution. The observed decrease was from 1.4 nm for the initial growth of 50 nm down to 0.5 nm after the total growth of a 200 nm thick layer.

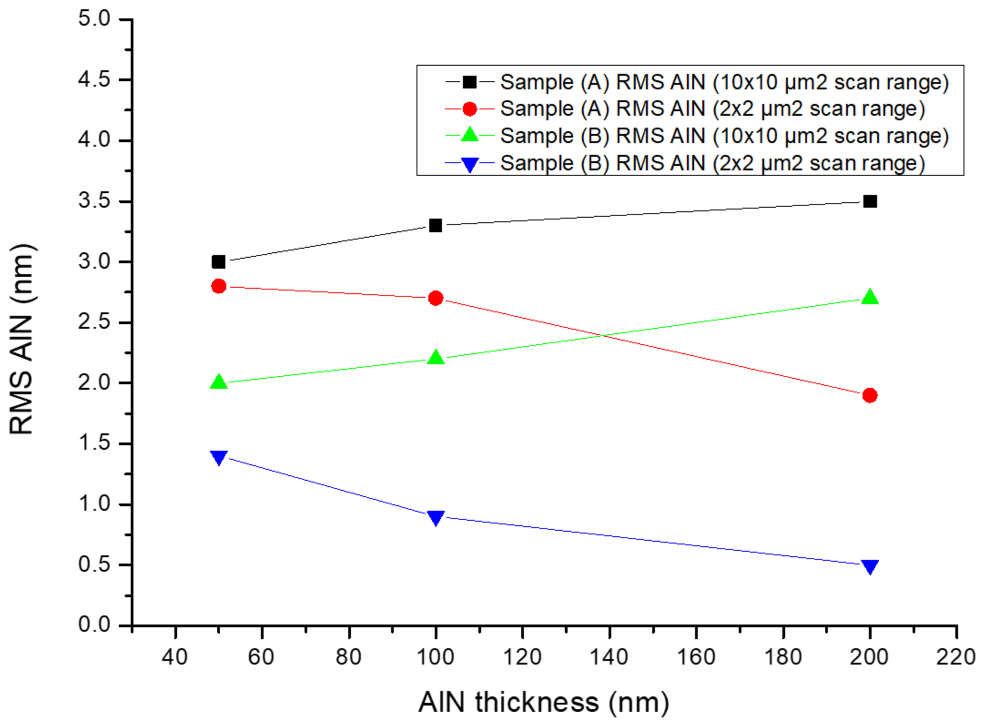

Figure S2. RMS roughness values evaluated as a function of the AlN thickness for both sample A

and sample B. (a) represents the variation in RMS roughness for sample A, while (b) shows the variation for sample B. The RMS values were measured on atomic force mi-croscopy topographic images of size (10 x 10) µm^2 and (2 x 2) µm^2.

3. Photoluminescence properties of $Al_{0.3}Ga_{0.7}N$ QDs

Regarding the PL properties of $Al_{0.3}Ga_{0.7}N$ (n.c.) QDs in $Al_{0.7}Ga_{0.3}N$ cladding layers, the PL integrated intensity was compared to a reference structure based on a QD plane grown on sapphire. In this case, the QDs are made of $Al_{0.1}Ga_{0.9}N$ (n.c.) QDs in $Al_{0.5}Ga_{0.5}N$ cladding layers and therefore emit at a higher wavelength, but the comparison has been done to investigate more deeply the radiative efficiency of QDs grown on h-BN templates. Also, the PL intensity has been corrected based on the detection and optical fiber sensitivities as a function of the emitted wavelength. It has been found that the PL integrated intensity of the QDs grown on h-BN templates (samples A and B) is lower by 15 to 45 times compared to a reference structure based on QDs grown on sapphire. In addition, the intensity ratio of 3 between the two structures grown on h-BN indicates that the optimization of the h-BN template as well as the nucleation and growth conditions for (Al, Ga)N growth on h-BN could be further investigated and optimized to reach values as high, or even higher, as (Al, Ga)N growth on sapphire.

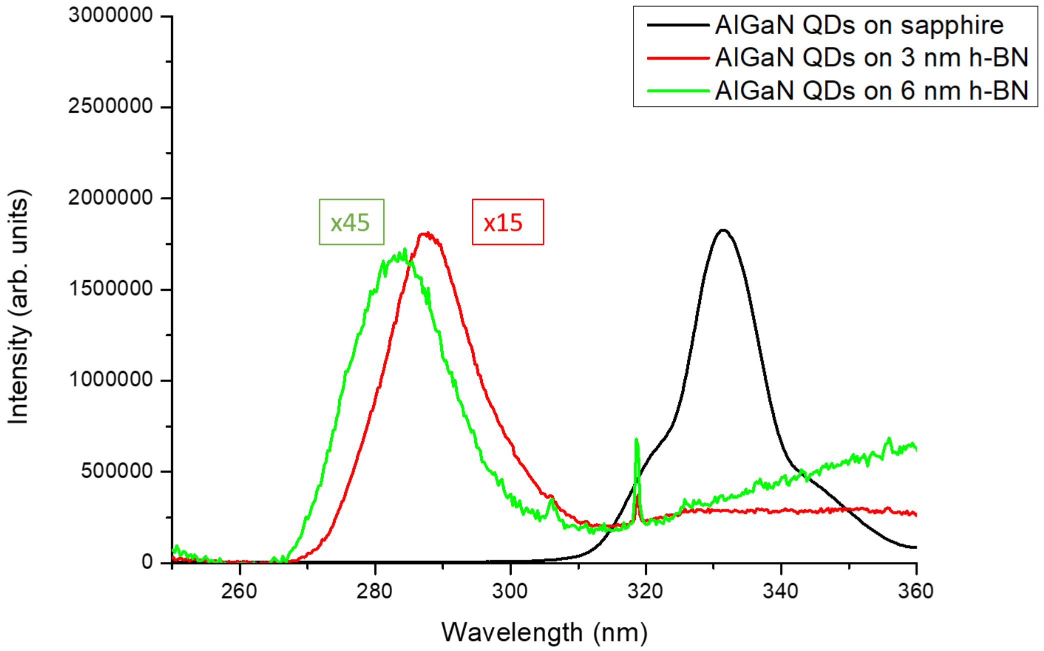

Figure S3. Photoluminescence measurements of an $Al_yGa_{1-y}N$ QD plane at room temperature (300K) for: a reference sample with $Al_{0.1}Ga_{0.9}N$ (n.c.) QDs / $Al_{0.5}Ga_{0.5}N$ grown on sapphire (black

color), a sample with $Al_{0.3}Ga_{0.7}N$ (n.c.) QDs / $Al_{0.7}Ga_{0.3}N$ grown on 3 nm h-BN template on sapphire (red color), and a sample with $Al_{0.3}Ga_{0.7}N$ (n.c.) / $Al_{0.7}Ga_{0.3}N$ QDs grown on 6 nm h-BN template on sapphire (green color). The spectra show that the PL integrated intensity is around 15 times (red spectrum) to 45 times (green spectrum) lower compared to the reference sample.

4. Photoluminescence properties of different $Al_yGa_{1-y}N$ QDs structures grown directly on sapphire and on h-BN / sapphire

Table S1 provides a comparison of the PL properties in terms of the PL integrated intensity ratio between LT and RT of $Al_{0.7}Ga_{0.3}N$ /$Al_{0.3}Ga_{0.7}N$ QDs structures grown on sapphire substrates and on h-BN / sapphire templates. It can be found that: 1) the best $Al_{0.3}Ga_{0.7}N$ QDs sample grown on sapphire (sample 2), which has a PL ratio of 1.4, compared to the PL ratio of the less efficient sample grown on h-BN (sample B), which has a PL ratio of 13, shows that the PL intensity between LT and RT drops approximately 10 times more in the case of AlGaN QDs grown on h-BN. 2) Secondly, when comparing the PL ratio of the least efficient sample grown on sapphire (sample 3) with a PL ratio of 6.9 to the PL ratio of the most efficient sample grown on h-BN (sample A) with a value of 7, it points out at a very similar drop of the PL intensity between LT and RT for both samples. Lastly, comparing the PL ratio of the best sample grown on sapphire (sample 2) with a PL ratio of 1.4 to the PL ratio of the best sample grown on h-BN (sample A) with a PL ratio of 7, it shows that the PL intensity for the QDs structure grown on h-BN decreases 5 times faster only than for the best QDs sample directly grown on sapphire. Therefore, the difference is found to be limited compared to the important structural quality difference between the samples grown on h-BN and the samples directly grown on sapphire as shown in table S2 below.

Table S1. Wavelength emission and PL integrated intensity ratio between LT and RT for different $Al_yGa_{1-y}N$ QDs / $Al_xGa_{1-x}N$ structures grown on sapphire and on h-BN.

	Sample 1 (grown on sapphire)	Sample 2 (grown on sapphire)	Sample 3 (grown on sapphire)	Sample A (grown on h-BN/ sapphire)	Sample B (grown on h-BN/ sapphire)
	$Al_{0.7}Ga_{0.3}N$ /$Al_{0.3}Ga_{0.7}N$ QDs	$Al_{0.7}Ga_{0.3}N$ /$Al_{0.3}Ga_{0.7}N$ QDs	$Al_{0.7}Ga_{0.3}N$ /$Al_{0.3}Ga_{0.7}N$ QDs	$Al_{0.7}Ga_{0.3}N$ /$Al_{0.3}Ga_{0.7}N$ QDs / 3nm h-BN	$Al_{0.7}Ga_{0.3}N$ /$Al_{0.3}Ga_{0.7}N$ QDs / 6 nm h-BN
Wavelength (nm) at 12K	292	294	298	275	279
Wavelength (nm) at 300K	300	301	306	275	280
PL Integrated intensity ratio (LT/RT)	2.0	1.4	6.9	7	13

Table S2. X-ray rocking curve diffraction measurements of (0 0 0 2) and (1 0 −1 3) symmetric and asymmetric reflections for $Al_{0.7}Ga_{0.3}N$ structures grown on sapphire and $Al_{0.7}Ga_{0.3}N$ structures grown on h- BN / sapphire.

	FWHM	
	(0 0 0 2) (°)	(1 0 −1 3) (°)
$Al_{0.7}Ga_{0.3}N$/AlN/Sapphire structure (1)	0.3	0.4
$Al_{0.7}Ga_{0.3}N$/AlN/Sapphire structure (2)	0.3	0.5
$Al_{0.7}Ga_{0.3}N$/AlN/Sapphire structure (3)	0.5	0.6
$Al_{0.7}Ga_{0.3}N$/AlN/3 nm h-BN/Sapphire (Sample A)	1.1	6.6
$Al_{0.7}Ga_{0.3}N$/AlN/6 nm h-BN/Sapphire (Sample B)	0.8	3.3

This property clearly emphasizes the advantage of using QDs on high defect densities materials obtained through simple and monolithic growth processes. Indeed, such processes could lead to cost effective approaches, which is a very important feature for the use of UV LEDs in daily life applications.
